# A "trained immunity" inducer-adjuvanted nanovaccine reverses the growth of established tumors in mice

**DOI:** 10.1186/s12951-023-01832-3

**Published:** 2023-03-02

**Authors:** Duo Li, Weiran Li, Peng Zheng, Ying Yang, Qingwen Liu, Yongmao Hu, Jinrong He, Qiong Long, Yanbing Ma

**Affiliations:** 1 Laboratory of Molecular Immunology, Institute of Medical Biology, Chinese Academy of Medical Sciences and Peking Union Medical College, Kunming, 650118 China; 2 Department of Acute Infectious Diseases Control and Prevention, Yunnan Provincial Center for Disease Control and Prevention, Kunming, China; 3 Institute of Medical Biology, Kunming Medical University, Kunming, China; 4 School of Life Sciences, Yunnan University, Kunming, China

**Keywords:** Nanovaccine, Trained immunity, MDP, β-glucan, Tumor

## Abstract

Innate immune cells are critical in antitumor immune surveillance and the development of antitumor adaptive cellular immunity. Trained innate immune cells demonstrate immune memory-like characteristics, producing more vigorous immune responses to secondary homologous or heterologous stimuli. This study aimed to investigate whether inducing trained immunity is beneficial when using a tumor vaccine to promote antitumor adaptive immune responses. A biphasic delivery system was developed with the trained immunity inducer Muramyl Dipeptide (MDP) and specific tumor antigen human papillomavirus (HPV) E7 peptide encapsulated by poly(lactide-co-glycolide)-acid(PLGA) nanoparticles (NPs), and the NPs along with another trained immunity agonist, β-glucan, were further embedded in a sodium alginate hydrogel. The nanovaccine formulation demonstrated a depot effect for E7 at the injection site and targeted delivery to the lymph nodes and dendritic cells (DCs). The antigen uptake and maturation of DCs were significantly promoted. A trained immunity phenotype, characterized by increased production of IL-1β, IL-6, and TNF-α, was induced in vitro and in vivo in response to secondary homologous or heterologous stimulation. Furthermore, prior innate immune training enhanced the antigen-specific INF-γ-expressing immune cell response elicited by subsequent stimulation with the nanovaccine. Immunization with the nanovaccine completely inhibited the growth of TC-1 tumors and even abolished established tumors in mice. Mechanistically, the inclusion of β-glucan and MDP significantly enhanced the responses of tumor-specific effector adaptive immune cells. The results strongly suggest that the controlled release and targeted delivery of an antigen and trained immunity inducers with an NP/hydrogel biphasic system can elicit robust adaptive immunity, which provides a promising tumor vaccination strategy.

**Graphical Abstract:**

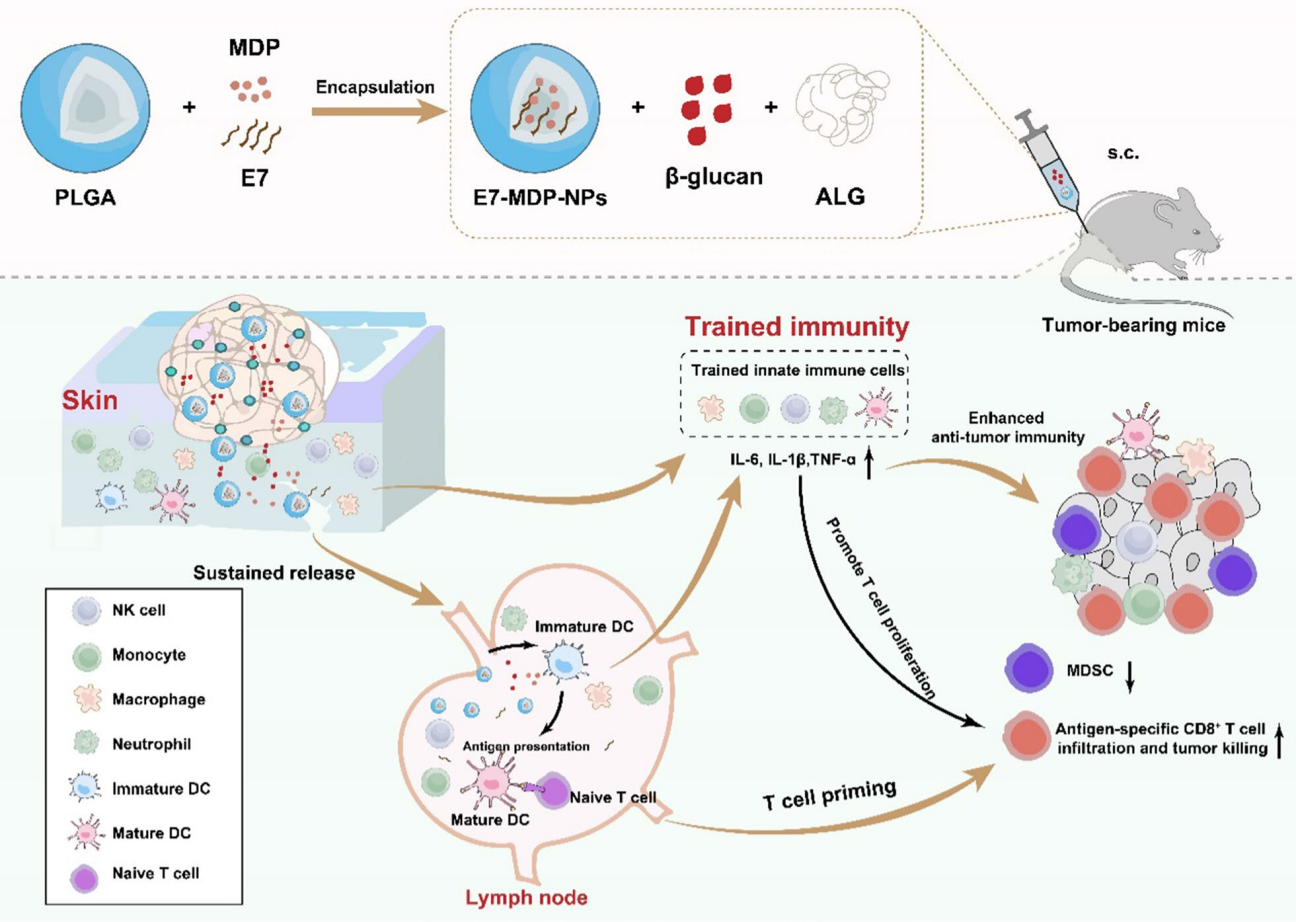

## Introduction

The innate immune system recognizes pathogens or endogenous danger signals through pattern recognition receptors (PRRs) [1]; it acts as a rapid first line of defense to fight microbial infections and functions in concert with adaptive immunity to develop long-lasting protective immunity [2]. Although the historical conventional view has been that only adaptive immunity can build immune memory, this theory has been challenged recently. Accumulated data have shown that innate immune cells undergo long-term transcriptomic, metabolic and epigenetic rewiring to adjust their functional program and display memory-like characteristics in a process termed ‘‘trained immunity’’ [3]. Innate immune cells are trained in response to specific initial stimuli, and these trained cells produce a more rapid and stronger response to secondary homologous or even heterologous stimuli, indicating enhanced immune responses, such as proinflammatory cytokine and ROS expression [4].

Most of the knowledge on trained immunity is derived from studies with the Bacillus Calmette-Guerin (BCG) vaccine. It has been well documented that BCG vaccination reduces neonatal all-cause mortality, especially that due to lethal infections, such as neonatal sepsis and respiratory infection [5]. The nonspecific protective effects of BCG found in infants have also manifested in adults [6, 7]. BCG vaccination causes enhanced heterologous cytokine secretion and epigenetic reprogramming of inflammatory cytokine genes, leading to reduced viremia and increased protection against viral infections in human studies [7] and experimental studies performed with murine models [8]. In addition to BCG, certain vaccines, such as the polybacterial mucosal vaccine MV130 [9], measles vaccine [10], smallpox vaccine, oral polio vaccine (OPV), influenza vaccine [11, 12] and even mRNA vaccines against coronavirus disease 2019 (COVID-19) [13], have been shown to induce trained immunity signatures, providing nonspecific protection against unrelated infections and improving morbidity and mortality [14, 15]. Further studies have detailed that certain microbial components have the capacity to trigger trained immunity and confer protection against heterologous lethal infections [16, 17]. The typical agonists of trained immunity include β-glucan, a fungal cell wall polysaccharide; flagellin, a component of bacterial flagella; muramyl dipeptide (MDP), a bacterial cell wall peptidoglycan component; chitin, a fungal cell wall component; lipopolysaccharide (LPS), a membrane component of gram-negative bacteria; and the synthetic oligodeoxynucleotide analogs poly(IC:LC) and CpG [17, 18]. It has been proven that innate immune memory can last for several months to more than 1 year and that live attenuated vaccines can lead to heterologous protection against infections for up to 5 years or even a decade [19, 20].

Trained immunity agonists are also known to have antitumor activity. For example, BCG is widely used to treat bladder cancer [21]. The well-trained immune response induced by BCG vaccination was shown to be dependent on the binding of the peptidoglycan component MDP [22] to the intracellular receptor nucleotide-binding oligomerization domain 2 (NOD2). In addition, the NOD2 agonist mifamurtide has been approved for use as an immunotherapy in patients with osteosarcoma in combination with chemotherapy following complete surgical resection of the primary tumor. β-glucan is an immunomodulatory agent that acts as a pathogen-associated molecular pattern (PAMP) recognized by pattern recognition receptors (PRRs) expressed on innate immune cells, and it has been associated with tumor immunotherapy efficacy [23]. β-glucan signaling occurs through dectin-1 receptors on the cell surface, which activate intracellular signaling cascades that drive trained immune responses [24, 25]. In addition to binding to the receptor dectin-1, β-glucan has been suggested to utilize other receptors to induce trained immunity.

Trained immunity phenotypes have been described in hematopoietic stem cells (HSCs), monocytes, macrophages, neutrophils, natural killer (NK) cells, innate lymphoid cells (ILCs) and dendritic cells (DCs), as well as in nonimmune cells such as endothelial and epithelial cells [16, 26]. The epigenetic marks with increased levels in trained cells include H3K4me3, H3K27ac, and H3K4me1, which are associated with open promoter regions and enhancer sites [22, 27] of genes related to inflammatory responses and immunological signaling [7, 28], while the level of the repressor mark H3K9me3, which is associated with closed chromatin, decreased [3, 29]. Another key mechanism of trained immunity responses is metabolic reprogramming through a shift from oxidative phosphorylation to aerobic glycolysis mediated by the Akt/mTOR/HIF-1α pathway [30]. Typically, trained immunity favors the production and release of proinflammatory cytokines, such as TNF-α, IL-6 and IL-1β by innate immune cells upon exposure to a second stimulus [31, 32].

Of note, while trained immunity provides nonspecific protection through innate immunity, it also enhances antigen-specific adaptive T-cell responses [33, 34]. The supporting evidence includes the observations of massive accumulation of tumor-specific T cells in urine after successful BCG therapy for bladder cancer [35], enhanced specific antibody responses to influenza A vaccination and cytokine production after vaccination with BCG [36], efficient immunity and specific antibody responses against SARS-CoV-2 variants induced by a BCG-adjuvanted spike antigen [37], and an enhancement in the T-cell response to unrelated influenza antigens [38] after vaccination with the sublingual vaccine MV130. It is feasible for trained cells to mediate a long-lasting effect that enhances T-cell responses for up to 1 year [39]. DCs, as the key cellular link between innate and adaptive immunity [40], play a pivotal role in enhancing adaptive T-cell responses [39, 41]. Trained DCs with strong immunostimulatory properties were reported to be characterized by increased expression of certain PRRs and the release of typical innate immune cytokines, such as IL-1β.

This study proposed that taking advantage of the “memory” properties of trained immunity might explore a new avenue for eliciting robust and long-lasting antitumor adaptive immune responses in designing an efficient tumor vaccine. In this study, a ‘‘two-phase releas’’ delivery system-based nanovaccine was developed, with the tumor antigen human papillomavirus 16 (HPV16) E7 peptide and MDP encapsulated in PLGA nanoparticles (NPs) and further embedded along with β-glucan in a sodium alginate (ALG) hydrogel. The capacity of the formulation to induce trained immunity was assessed in vitro and in vivo*,* and its antitumor effects were demonstrated in TC-1 tumor mouse models.

## Materials and methods

### Mice and cells

C57BL/6 mice (6–8 weeks old) were provided by the Institute of Medical Biology, Chinese Academy of Medical Sciences (CAMS), and maintained in a specific pathogen-free facility. TC-1 tumor cells, which were obtained by cotransformation of HPV16 E6 and E7 and the activated ras oncogene into primary C57BL/6 mouse lung epithelial cells [42], were grown in RPMI 1640 medium supplemented with 10% (vol/vol) fetal calf serum (FCS). The protocols used for animal experiments were approved by the Animal Care and Welfare Ethics Committee of the Institute of Medical Biology (license number: SYXK (dian) K2019-0003).

### Preparation of nanovaccines

The innate immune stimulator MDP (Sigma‒Aldrich) and/or the specific E7 epitope peptide were encapsulated into PLGA NPs by a two-stage emulsification method as described previously [43]. In brief, 0.5 mg of E7 peptide (^44^QAEPDRAHYNIVTFCCKCD^62^) or FITC-labeled E7 peptide (FITC-E7), which were each synthesized by Sangon Biotech (Shanghai), and/or 1.5 mg of MDP was added to 3 mL of dichloromethane (DCM) (Jinan Daigang Biomaterials Co., Ltd.) containing 90 mg of PLGA. The mixed solution was sonicated for 1 min to produce a primary water-in-oil emulsion, mixed with 12 mL of 2% (w/v) PVA and further sonicated for 5 min to form a secondary water–oil-water emulsion. After stirring overnight to completely evaporate the DCM, NPs were obtained by centrifuging the emulsion at 21,000 ×*g* for 30 min, washing with distilled water 3 times, and resuspending the pellet in phosphate-buffered saline (PBS). The PLGA NPs were named MDP-NPs, E7-NPs, and E7-MDP-NPs, depending on the encapsulated contents.

Hydrogels were prepared by dissolving β-glucan (InvivoGen) in ALG (10 mg/mL ALG solution) (Sigma‒Aldrich) and NPs to prepare β-glucan@ALG, MDP-NPs + β-glucan@ALG, E7-NPs + β-glucan@ALG and E7-MDP-NPs + β-glucan@ALG.

### Characterization of PLGA NPs

NP samples were fixed with 2.5% cold glutaraldehyde in 0.2 M sodium cacodylate buffer (pH 7.4) for 2 h at 4 °C, followed by incubation in % osmium tetroxide in 0.1 M sodium cacodylate buffer (pH 7.4) at 4 °C for 1 h. The samples were observed and imaged with a transmission electron microscope (TEM, Hitachi).

Dynamic light scattering (DLS) measurements were performed using a Zetasizer Nano ZS (Malvern Instruments) to determine the NP size distribution and polydispersity index (PDI), with PDI values ranging from 0.0 (monodispersed) to 1.0 (completely heterodispersed).

The content of E7 or MDP in the supernatants of NP preparations, i.e., the content that was not encapsulated into NPs, was determined with a Micro BCA kit [43] for E7 and with a colorimetric assay to detect N-acetyl-glucosamine-[44] for MDP.

### Drug release from PLGA NPs

E7-MDP-NPs and E7-MDP-NPs@ALG were placed in dialysis bags (molecular weight cutoff value, 7 kDa; Biosharp) and dialyzed against PBS or dulbecco's modified eagle medium (DMEM) with 10% fetal bovine serum (FBS). Dialysis was performed at 37 °C with gentle stirring. The content of the E7 peptide and MDP in the dialysis buffer was determined as described above.

### Biodistribution and duration of the tumor antigen in vivo

To observe the residence time of the tumor antigen E7 encapsulated in NPs and the hydrogel at the injection site, 2 μg of free FITC-E7 or 1 mg of FITC-E7-NP, as well as its mixture with the ALG solution, was subcutaneously injected into the back of C57BL/6 mice, and the fluorescence signal of FITC-E7 at the injection site was detected through live imaging at different timepoints with an In-Vivo FX PRO imaging system (Bruker).

To analyze the in vivo biodistribution of E7 and its migration to the draining lymph nodes, six hours after injection, major organs, including the heart, liver, spleen, lungs, kidneys, and inguinal lymph nodes, were obtained from sacrificed mice for ex vivo FITC fluorescence imaging. Fluorescence imaging was evaluated with Bruker MI SE analysis software (Bruker).

### Bone marrow-derived DC (BMDC) maturation

BMDCs were obtained from the tibia and fibula of male C57BL/6 mice as described previously [43, 45] and seeded at 1 × 10^6^ cells/well in 96-well plates. Different concentrations of E7-NPs, E7-MDP-NPs, and E7-MDP-NPs + β-glucan were added to the cultures. After incubating for 24 h, 10 µl of CCK-8 solution was added to each well, and the OD450 value was recorded after a 4 h incubation to detect cell viability.

The activation and maturation of BMDCs driven by different formulations were analyzed by detecting the expression of cell-surface markers on BMDCs. BMDCs were incubated with PBS, MDP (1, 10 and 100 ng), β-glucan (10, 50 and 100 μg), 100 μg of MDP-NPs, E7-NPs, E7-MDP-NPs, a mixture of 100 μg of E7-NPs with 100 μg of β-glucan, or a mixture of E7-MDP-NPs with 100 μg of β-glucan for 24 h. The surface marker expression of collected BMDCs was evaluated with flow cytometry using PE-conjugated anti-CD11c, FITC-conjugated anti-CD80, APC-conjugated anti-CD86, APC-conjugated anti-H-2 Kb (MHC I) and FITC-conjugated anti-IA/IE (MHC II) antibodies according to previously described methods [45]. Data were collected on a flow cytometer (BD FACSCanto II) and analyzed using FlowJo Software.

### Antigen uptake by BMDCs

To investigate the E7 peptide uptake efficiency of BMDCs mediated by NPs or β-glucan-assisted NPs, BMDCs were incubated with 0.2 μg of FITC-E7, 100 μg of FITC-E7-NPs or FITC-E7 -MDP-NPs, a mixture of 100 μg of FITC-E7-NPs with 100 μg of β-glucan, or a mixture of 100 μg of E7-MDP-NPs with 100 μg of β-glucan for 8 h, and then cells were harvested for flow cytometric analysis.

In a parallel experiment, BMDCs were labeled with 1 μg/mL DAPI nucleic acid staining solution and incubated at 37 °C for 10 min. After centrifugation at 500 ×*g* for 5 min to remove excess dye, images were acquired using a Leica TCS SP2 confocal microscope.

### Lysosomal escape assay in BMDCs

An lysosomal escape assay was conducted as previously described [46]. Briefly, BMDCs were treated with PBS, free FITC-E7, or FITC-E7-MDP-NPs for 0.5 h, 2 h, 4 h, and 6 h. After washing with PBS, lysosomes were stained with LysoTracker red (Beyotime) following the manufacturer’s protocol. After washing with PBS, the cells were observed by confocal microscopy (ImageXpress Micro Confocal, Molecular Devices).

### The activation of DCs and the in lymph nodes

The maturation of DCs in the body was detected as described previously [45]. Briefly, 2 μg of free FITC-E7 or 1 mg of FITC-E7-MDP-NPs was subcutaneously injected into mice. After 24 h, the lymph nodes were isolated, dispersed, and passed through a 40 μm membrane to prepare single-cell suspensions. Cells were then washed, blocked, and subjected to flow cytometry staining with anti-CD11c, CD80, CD86, MHC I and MHC II antibodies. Data were then collected in a CytoFLEX flow cytometer (Beckman Coulter).

In addition, the cells were stained with anti-CD103, CD11b, and CD8α antibodies, and then the cells gated with FITC^+^ were further analyzed for CD103^+^cells, CD11b^+^ CD8α^−^, and CD8α^+^cells by flow cytometry to clarify the proportion of the migrated DCs and resident DCs in the lymph nodes**.**

### Characterization of in vitro training with peripheral blood mononuclear cells (PBMCs)

8 week-old female C57BL/6 mice were anesthetized using vaporizer inhalation of 5% isoflurane, and PBMCs were isolated by Percoll (Solarbio) discontinuous density gradient centrifugation after cardiac blood sampling.

PBMCs (5 × 10^5^/mL) were added to a flat-bottom 96-well plate and incubated with PBS, 100 μg of E7-NPs, 100 μg of E7-NPs + 0.2 μg of β-glucan, 100 μg of E7-MDP-NPs, or 100 μg of E7-MDP-NPs + 0.2 μg of β-glucan for 24 h at 37 °C. The supernatant was discarded, and the cells were washed and incubated with fresh RPMI medium supplemented with 10% fetal bovine serum. The medium was changed after 3 days of culture at 37 °C, and the cells were stimulated with 10 ng/mL LPS for an additional 24 h after a medium change on day 7. Subsequently, the supernatant was stored at − 20 °C until ELISA was performed.

### Ex vivo evaluation of in vivo trained immunity

For in vivo trained immunity immunization experiments, 8 week-old female C57BL/6 mice were inoculated with PBS or different combinations of nanovaccine formulations by the subcutaneous route, and 4 weeks later, 10 μg/mouse LPS was administered by intraperitoneal injection. After 4 h, blood was collected and centrifuged at 100 ×*g* for 10 min to obtain the serum for detection of cytokine levels.

To investigate the effect of inducing trained immunity on the promotion of the antigen-specific adaptive immune response elicited by the nanovaccine, 8 week-old female C57BL/6 mice were inoculated subcutaneously with NPs or MDP-NPs + β-glucan, followed by stimulation with the nanovaccine formulation E7-MDP-NPs + β-glucan@ALG 4 weeks later. PBS was administered as a control. After 1 week, splenocytes were isolated to detect the antigen-specific response of IFN-γ-expressing lymphocytes with an ELISpot assay.

### Cytokine measurement and ELISpot assay

The concentrations of cytokines in the supernatants of murine PBMCs or mouse sera were assayed using commercial ELISA kits for IL-1β, IL-6 and TNFα (Abcam) according to the manufacturer's instructions.

IFN-γ-expressing splenocytes were analyzed as described previously [47] using a kit purchased from Dakewe Biotechnology Co., Ltd. (China).

### Tumor models and immunization

The establishment of an HPV-related mouse xenograft tumor model was performed according to previous procedures [43]. For preventive studies, mice were administered 100 μL of PBS, 1 mg of MDP-NPs, 1 mg of MDP-NPs + 1 mg of β-glucan, 1 mg of E7-NPs, 1 mg of E7-MDP-NPs, 1 mg of E7-NPs + 1 mg of β-glucan@ALG, or 1 mg of E7-MDP-NPs + 1 mg of β-glucan@ALG subcutaneously on days 0, 7 and 14, and then 1 × 10^5^ TC-1 cells mixed with an equal volume of Matrigel (BD) were inoculated subcutaneously in the right back region of mice on day 21. Tumor size was measured periodically with a Vernier caliper.

For therapeutic studies, mice were first inoculated with TC-1 cells to form tumors, and when the tumors reached approximately 2–4 mm in diameter, immunizations with nanovaccine formulations were performed 3 times with a 1 week interval.

### Flow cytometric analysis

Immune cells were isolated from mouse spleen tissue using a mouse spleen lymphocyte isolation kit (Solarbio), and a single-cell suspension was prepared for cytotoxic T lymphocyte (CTL) and myeloid-derived suppressor cell (MDSC) counting and fluorophore-conjugated antibody labeling. Fluorescent dye-labeled antibodies were obtained from BioLegend (San Diego, CA, USA). For analysis of CTLs, APC-conjugated anti-CD8α and PE-conjugated anti-IFN-γ were used. PE-conjugated anti-Gr-1 and APC-conjugated anti-CD11b were used for MDSC analysis. Cells were analyzed by flow cytometry, and data were analyzed using FlowJo software.

### Statistical analysis

All values are reported as the mean ± standard error of the mean (SEM). Data were evaluated by one-way or two-way analysis of variance (ANOVA) using GraphPad Prism 8 software to compare multiple groups.

## Results

### Characterization of NPs

**Fig. 1:**
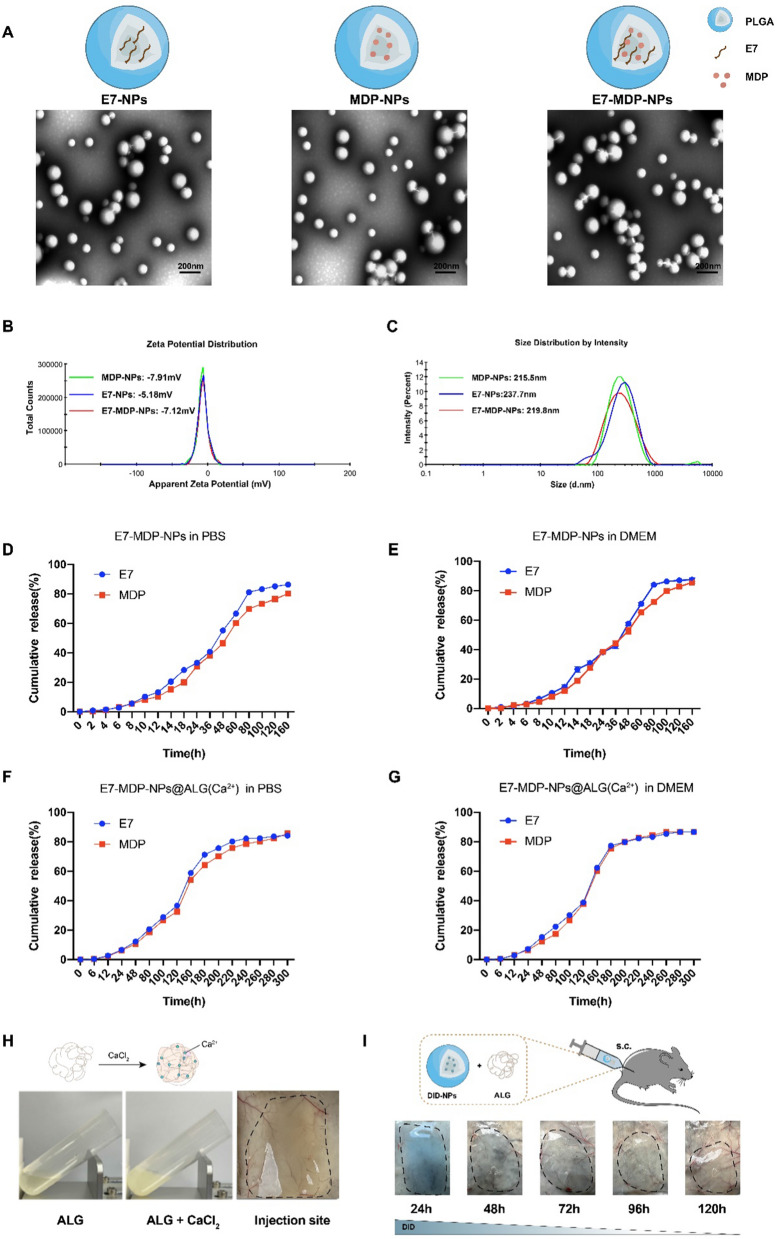
Characterization of nanoparticles. **A** TEM images of E7-NPs, MDP-NPs, and E7-MDP-NPs. High magnification, scale bar = 500 nm. **B** Average size distributions of the nanoparticles detected by DLS. **C** Average zeta potentials of the nanoparticles detected by DLS. **D**–G E7 and MDP release profiles of the nanoparticle-encapsulated E7 and MDP in PBS and DMEM medium with 10% FBS in vitro. **H** The formation of E7-MDP-NPs@ALG hydrogel in vitro* and *in vivo at 1 h after the injection. **I** Characterizations of the formation and persistence of E7-MDP-NPs@ALG hydrogel at the injection site in vivo

Prepared E7-NPs, MDP-NPs and E7-MDP-NPs were observed under a TEM, and morphologically, they appeared to be spherical particles. The three particles showed similar appearances, with diameters ranging from 100 to 300 nm (Fig. 1A). DLS analysis showed that the average diameters of E7-NPs, MDP-NPs, and E7-MDP-NPs were 237.7 nm with a PDI of 0.243, 215.5 with a PDI of 0.211 and 219.8 nm with a PDI of 0.229, respectively, and the zeta potentials were − 5.81 mV, − 7.91 mV, and − 7.12 mV, respectively (Fig. 1B, C). The encapsulated contents of the E7 peptide and MDP in E7-NPs, MDP-NPs and E7-MDP-NPs were determined by an indirect quantitative method that detects the content in the supernatant. The results showed that 1 mg of E7-NPs contained 2.19 ± 0.2 μg of E7 peptide, 1 mg of MDP-NPs contained 2.63 ± 0.2 μg of MDP, and E7-MDP-NPs contained 2.19 ± 0.2 μg of E7 and 2.12 ± 0.2 μg of MDP (Table 1).

**Table 1:** Mean size and antigen content of nanoparticles

Nanoparticle	Polydispersity	E7 Antigen content(μg/mg)	MDP content (μg/mg)
E7-NPs	0.243	2.19 ± 0.2	–
MDP-NPs	0.211	–	3.63 + 0.2
E7-MDP-NPs	0.229	2.19 ± 0.2	2.12 ± 0.2

To construct a two-phase release system, in this study, NPs were further mixed with ALG solutions containing or not containing β-glucan. The formation of the hydrogel was driven by the interaction of ALG with calcium ions. An in vitro experiment was performed to assess the E7 and MDP release profiles from both E7-MDP-NPs and E7-MDP-NPs@ALG biphasic delivery system. PBS and cell culture medium DMEM with 10% FBS were used to mimic a biological environment to some extent*,* and the releasing kinetics were detected. E7 and MDP were released in a similar profile whether in E7-MDP-NPs or in E7-MDP-NPs/ALG (Fig. 1D–G). The results indicated that the prepared NPs were well loaded with the E7 peptide and MDP and the encapsulated components in the NPs displayed characteristics of controlled and efficient release.

To evaluate the hydrogel formation capacity of the prepared ALG formulation, an in vitro experiment with thorough mixing of a 10 mg/mL ALG solution with a 10 mg/mL calcium chloride solution was performed and clearly showed a form transition from liquid to gel; in addition, 1 h after the injection of the ALG formulation into mice, the formation of a hydrogel was found locally at the injection site (Fig. 1H). Further, in an in vivo experiment, DID dye-encapsulated nanoparticles@ALG was administrated into the mice simulating the immunization of the nanovaccine. The release of encapsulated components was implied by the changing of the color in the hydrogel, which gradually faded with the time goes (Fig. 1I).

### NPs in combination with MDP and/or β-glucan promote antigen uptake and maturation by BMDCs

**Fig. 2:**
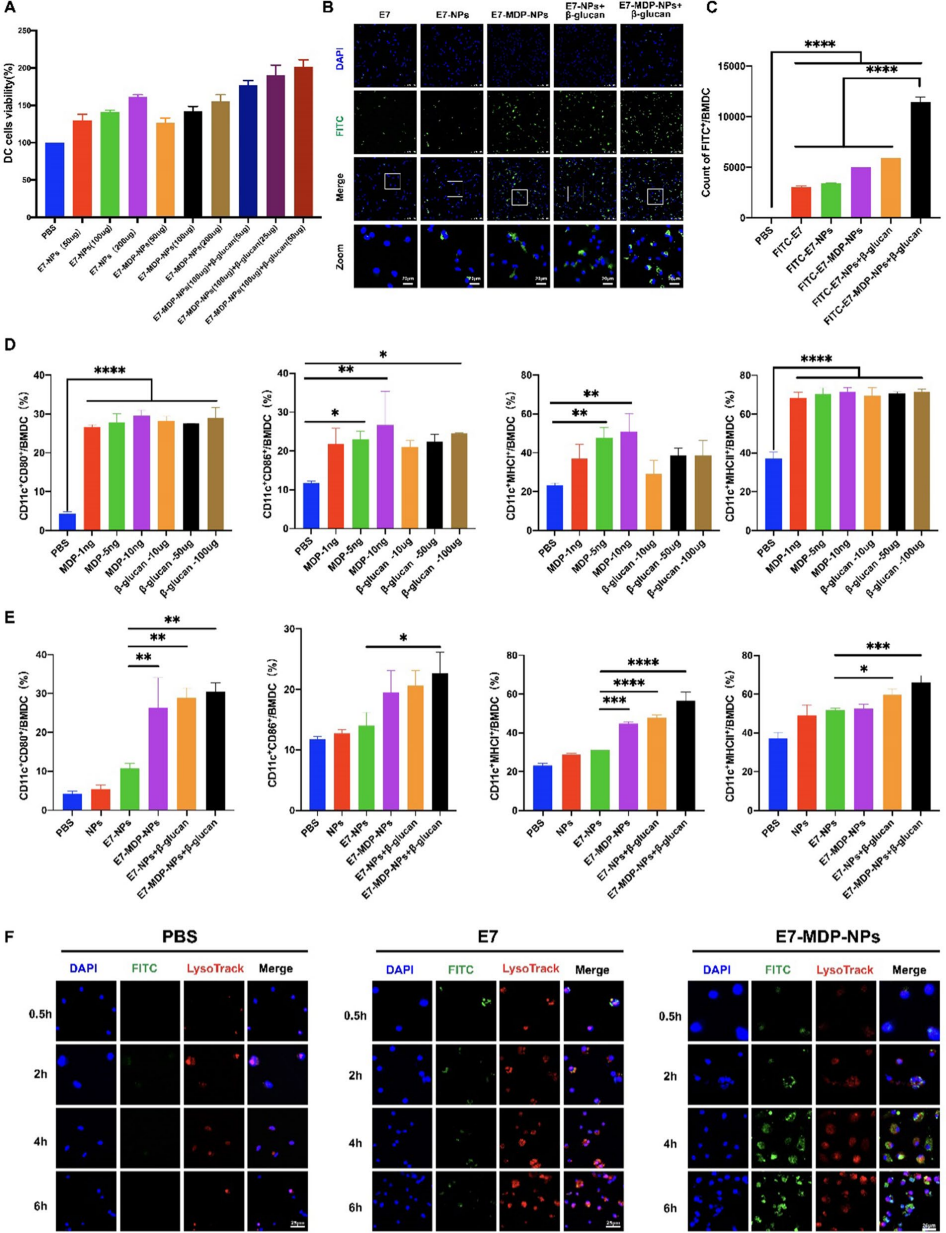
Nanoparticles in combination with MDP and/or β-glucan promote antigen uptake and maturation by BMDCs. **A** Viability of BMDCs after incubation with different nanovaccine formulations for 24 h. **B** Laser confocal microscopy analysis of antigen uptake by BMDCs. Cells were incubated with nanovaccines at 37 °C for 12 h. **C** FACS analysis of antigen uptake by BMDCs. Cells were incubated with nanovaccines at 37 °C for 8 h. **D** and **E** FACS analysis of the expression of the maturation markers CD80, CD86, MHC I, and MHC II. BMDCs were incubated with different concentrations of MDP or β-glucan or different formulations of nanovaccines for 24 h. **F** Lysosomal escape assay of E7-MDP-NPs in BMDCs. E7 was labeled with FITC (green), lysosomes were visualized with LysoTracker (red), and nuclei were stained with DAPI (blue).Data are presented as the mean ± SEM. *P < 0.05, **P < 0.01, ***P < 0.001; analyzed by one-way ANOVA with Tukey’s multiple comparisons posttest

First, whether the nanovaccine formulations were toxic to BMDCs was evaluated with a cell viability assay. BMDCs were incubated with different concentrations of E7-NPs, E7-MDP-NPs, and a mixture of E7-MDP-NPs with β-glucan for 24 h, and cell viability was detected by the CCK-8 method. The results showed that the different concentrations of the formulations did not show any cytotoxicity but rather exhibited a dose-dependent promotive effect on the cells (Fig. 2A).

To evaluate the efficiency of antigen uptake by BMDCs, cells were incubated with FITC-E7, FITC-E7-NPs, FITC-E7-MDP-NPs, FITC-E7-NPs + β-glucan or FITC-E7-MDP-NPs + β-glucan for 8 h. Laser confocal microscopy analysis showed that in comparison with free E7 peptide, the encapsulation of E7 peptides in PLGA NPs (E7-NPs) enhanced E7 uptake by BMDCs; furthermore, the inclusion of MDP and/or β-glucan increased the uptake efficiency (Fig. 2B). A more quantitative analysis was performed with flow cytometry, and significantly, the formulation of E7-MDP-NPs + β-glucan showed the highest uptake efficiency for E7 compared to the free E7 control and the other nanovaccine formulations (Fig. 2C). The above results indicated that β-glucan and MDP had the capability to promote uptake by BMDCs.

To further assess the effects of MDP, β-glucan, and the nanovaccines on BMDC maturation, the expression levels of CD80, CD86, MHC I and MHC II on the cell surface were analyzed by flow cytometry after an incubation for 24 h. Compared with PBS treatment, stimulation with MDP or β-glucan significantly promoted the expression of surface markers on mature BMDCs (Fig. 2D). In addition, the combined use of MDP or β-glucan with E7-NPs clearly enhanced the expression of mature BMDC surface markers compared to E7-NPs alone, while the formulation E7-MDP-NPs + β-glucan showed a more significant increase in expression (Fig. 2E). These results suggested that β-glucan and MDP were able to effectively activate BMDCs.

In addition, the endosomal/lysosomal escape capacity of E7-MDP-NPs in BMDCs was assessed. FITC-E7 peptide was up-taken by BMDCs at a lower efficiency, and E7 peptide (green) escaped from endosomal/lysosomal pathway was seldom seen while the E7 was mainly captured in the endosomal/lysosomal (yellow).In contrast, E7-encapsulted in nanoparticles was up-taken at a high efficiency, and more E7 escaped from endosomal/lysosomal as the incubation time increased. The results indicated that E7-MDP-NPs had significantly lysosomal escape capacity (Fig. 2F).

### The two-phase delivery system promotes the sustained release of E7 at the injection site and its lymph node migration

We took advantage of the ability of ALG to form hydrogels through interactions with calcium ions and combined E7-MDP-NPs and β-glucan to form a NP and hydrogel-constituted two-phase delivery system nanovaccine (E7-MDP-NPs + β-glucan@ALG).

**Fig. 3:**
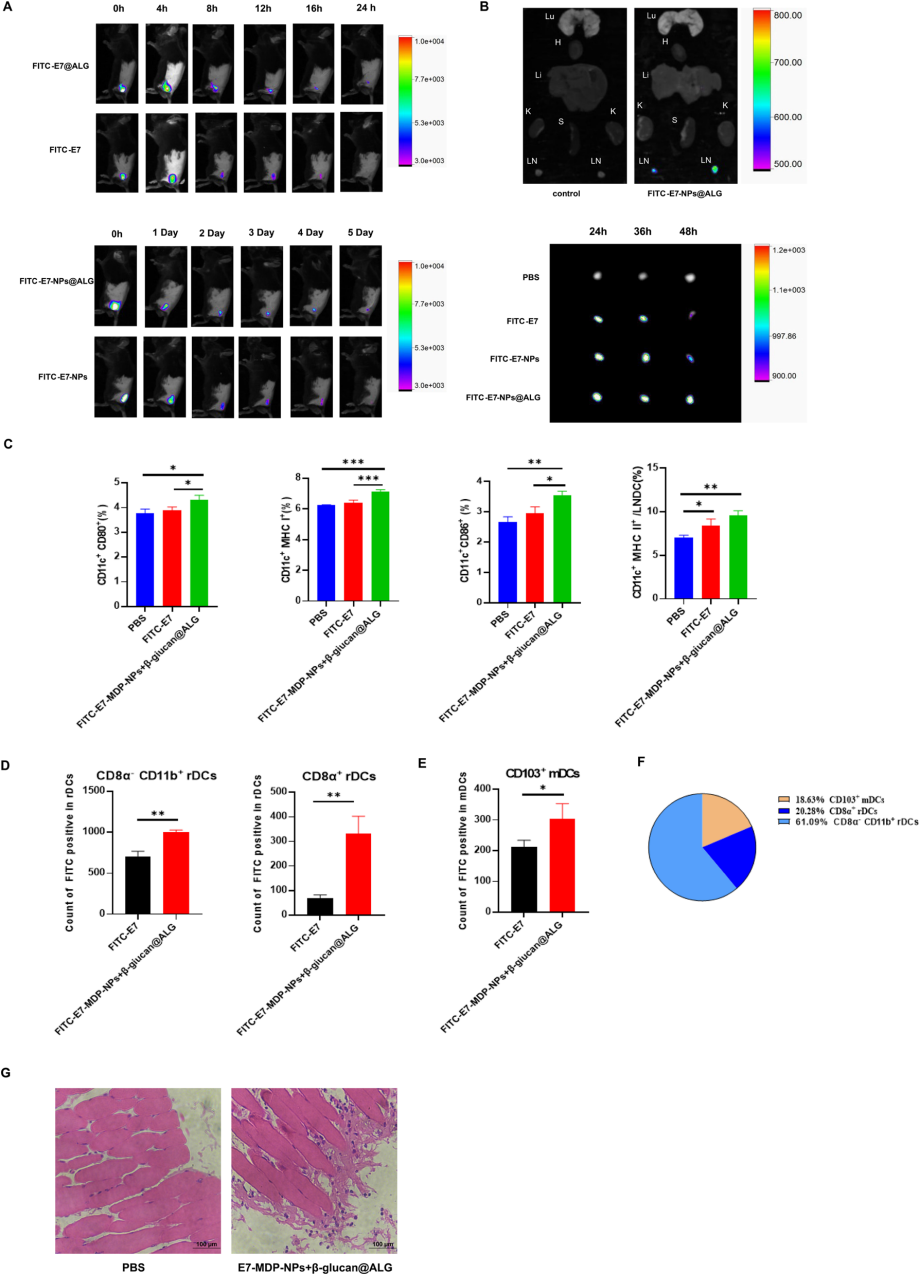
Sustained release and tracing of the E7 antigen delivered in different nanovaccine formulations in vivo. **A** Live imaging analysis of the release and residence of FITC-E7 at the injection site. **B** Ex vivo fluorescence images for analyzing the distribution of E7 in main organs (the heart (H), liver (Li), spleen (S), lungs (Lu), kidneys (K) and inguinal lymph nodes (LN)) and its accumulation in the inguinal lymph nodes. **C** Flow cytometry analysis on the expression of the maturation markers CD80, CD86, MHC I, and MHC II in DCs isolated from lymph nodes. **D** and **E** Flow cytometry analysis of CD8α^+^ rDCs, CD8α^−^CD11b^+^ rDCs, and CD103^+^ mDCs. The cells were isolated 24 h after the subcutaneous injection of free FITC-E7 and E7-MDP-NPs + β -glucan @ALG. **F** The proportion of the mDCs and rDCs in lymph nodes. **G** The infiltration of immune cells in the muscle tissue at the injection site, through histological analyses with H andE staining (Blue: nuclei; scale bar: 100 μm. D Data are presented as the mean ± SEM. *P < 0.05, **P < 0.01, ***P < 0.001; analyzed by one-way ANOVA with Tukey’s multiple comparisons posttest or T test

We clarified the characteristics of the controlled release and lymph node accumulation of the system-delivered antigen. First, free FITC-E7 was mixed with ALG to obtain FITC-E7@ALG, and then FITC-E7@ALG was subcutaneously injected into the back of mice, with free FITC-E7 used as a control. Live imaging results showed that E7 peptides delivered in the form of FITC-E7@ALG stayed at the injection site longer than free FITC-E7, as evidenced by the stronger fluorescence signal found in the FITC-E7@ALG group beginning at 12 h after the injection, while the fluorescence signal of free E7 was almost undetectable at 24 h (Fig. 3A, upper panel). The results indicated that the ALG hydrogel might improve the stability of E7 peptides or provide a feature necessary for controlled release.

Furthermore, FITC-E7-NPs and FITC-E7-NPs@ALG were subcutaneously inoculated into the back of mice to investigate the characteristics of the two-phase delivery system consisting of NPs and the hydrogel. Live imaging results showed that FITC-E7 NPs exhibited strong fluorescence at the injection site for 3 days, which was sustained for up to 5 days, compared to FITC-E7 (Fig. 3A, lower panel), while the signals for free FITC-E7 and FITC-E7@ALG were maintained for 16 h and 24 h, respectively. In addition, FITC-E7-NPs@ALG showed a stronger fluorescence signal than FITC-E7-NPs on day 4 and day 5 (Fig. 3A, lower panel). The results indicated that encapsulation by NPs dramatically prolonged the residence of the E7 antigen at the injection site, which may provide more persistent stimulation of the immune system; in addition, the application of the ALG hydrogel further improved the sustained release of E7 peptides.

In a parallel experiment, mice were sacrificed at 6 h after the injection of FITC-E7@ALG, and the heart, liver, spleen, lungs, kidneys, and inguinal lymph nodes were collected. Ex vivo fluorescence signals reflecting antigen accumulation were mainly observed in the lymph nodes (Fig. 3B, upper panel). Furthermore, after subcutaneous inoculation of free FITC-E7, FITC-E7-NPs or FITC-E7-NPs@ALG into the back of mice, the accumulation of fluorescence signals for labeled E7 in the lymph nodes of the mice was observed at different time points. The results showed that at 48 h, the fluorescence signal for FITC-E7-NPs@ALG remained strong, while that of FITC-E7-NPs decreased significantly and that of FITC-E7 was almost undetectable (Fig. 3B, lower panel), indicating that the combined use of NPs and the ALG hydrogel promoted the targeted migration and accumulation of the E7 peptide in the lymph nodes.

In addition, E7-MDP-NP + β -glucan@ALG effectively activated DCs in lymph nodes (Fig. 3C). Further, the resident DCs in LNs and circulatory DCs which transport back to LNs from the injection sites were analyzed by flow cytometry. The antigen loading was detected by FITC-E7, resident DCs (rDCs) was measured in cells with CD8α^−^CD11b^+^ and CD8α^+^ respectively to represent two subgroups (Fig. 3D), and the migrated DCs (mDCs) was detected by CD103^+^ (Fig. 3E). The proportions of the three groups of DCs in LNs isolated from the mice receiving E7-MDP-NPs + β -glucan @ALG were presented in a pie diagram ((Fig. 3F). We clarified that the antigen actively migrated into the lymph node and activated the resident DCs; and, there were circulatory DCs up-taking the antigen at the injection site and migrating in LNs, for both of which the nanoparticle-encapsulated E7 presented more efficiently than free E7. The proportion of resident DCs was far higher than.that of migrated DCs in LNs isolated from the mice receiving E7-MDP-NPs + β -glucan @ALG.

Compared with PBS group, E7- MDP-NPs + β -glucan @ALG induced the infiltration of a large number of immune cells into the injection site of mice (Fig. 3G), through analyzing the sections of the muscle tissue at the injection site with Hematoxylin and Eosin staining.

### Preventive immunizations with nanovaccines significantly prevent the growth of grafted TC-1 tumors

**Fig. 4:**
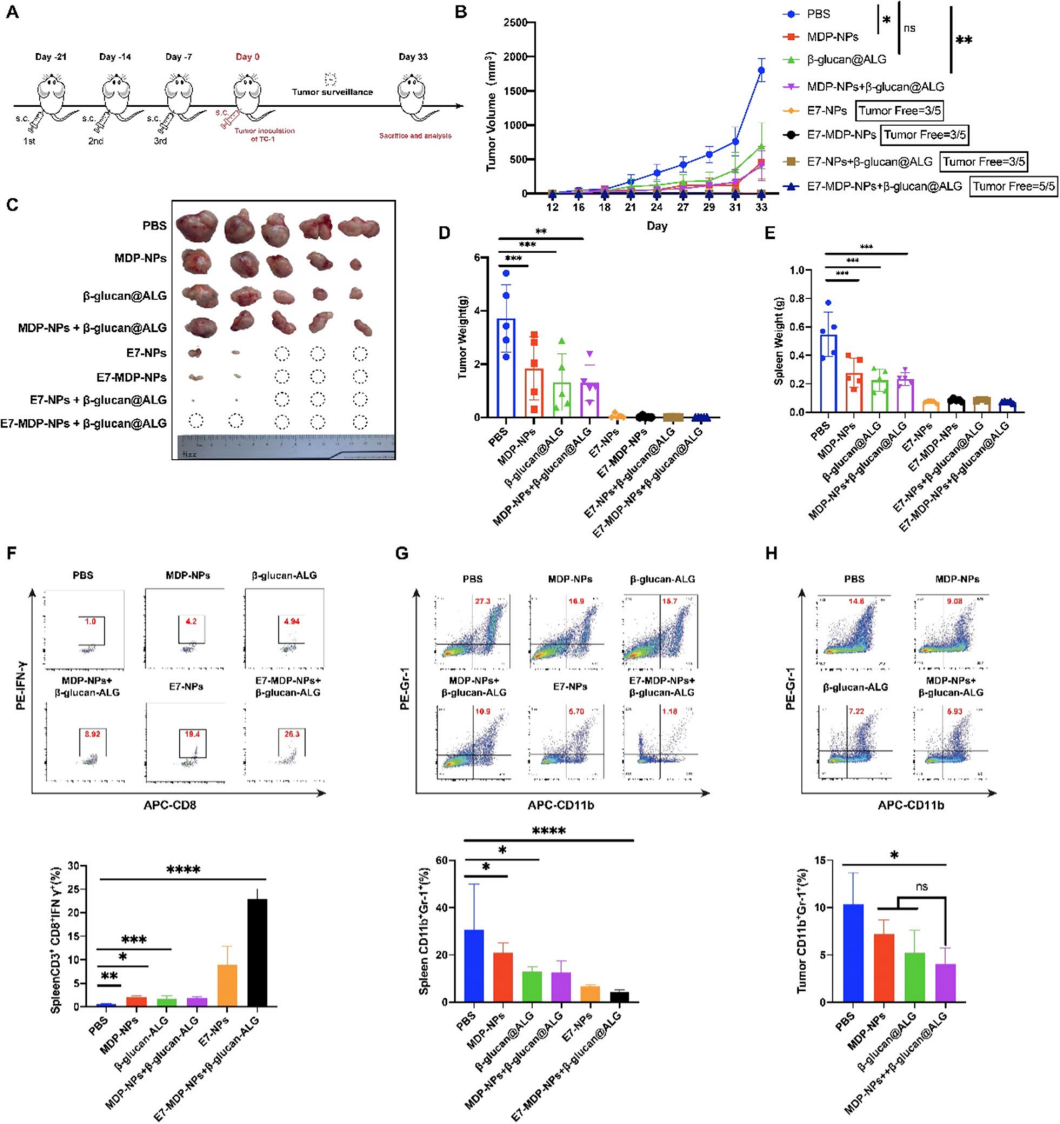
Preventive immunization with nanovaccines significantly suppressed the growth of subsequently inoculated TC-1 tumors in C57BL/6 mice. **A** The schedules for immunization with the nanovaccines and establishment of transplanted TC-1 tumors. n = 5 mice per group. **B** Dynamic monitoring of tumor growth. Mice were subjected to subcutaneous grafting of TC-1 tumor cells one week after the last immunization. **C** Representative images reflecting the sizes of collected tumor masses. **D** The weights of isolated tumor masses and (**E**) spleens. (**F**) Statistical data for analyzing IFN-γ-expressing CD3^+^CD8^+^ splenocytes. The splenocytes were stimulated with the E7 peptide and analyzed by FACS analysis. **G** Statistical data for analyzing the frequencies of CD11b^+^Gr-1^+^ cells in splenocytes and (**H**) tumor-infiltrating lymphocytes. Shown data were from two independent repetitive studies and analyzed by one-way ANOVA with Tukey’s multiple comparisons

To evaluate the potential of the nanovaccines and the important immunomodulatory components MDP and β-glucan to elicit antitumor immunity, the formulations were administered to C57BL/6 mice in advance, followed by subcutaneous transplantation of TC-1 cells (Fig. 4A). Compared to that in mice receiving PBS, the tumor growth in mice receiving MDP-NPs, β-glucan@ALG or MDP-NPs + β-glucan@ALG was significantly inhibited (Fig. 4B, C), indicating that MDP and β-glucan might regulate the immune system and improve antitumor immunity even in the absence of a specific tumor antigen. The inclusion of E7-NPs in different nanovaccines produced more significant suppression of tumor growth in comparison to the treatments without the E7 antigen, and noticeably, the E7-MDP-NPs + β-glucan@ALG group exhibited completely inhibited tumor growth and remained tumor free (Fig. 4C).

On day 43, tumors and spleens were removed from euthanized mice and weighed. The results for tumor weight (Fig. 4D) were consistent with the tumor growth curve (Fig. 4B) and tumor size results (Fig. 4C). Correspondingly, compared with the PBS group, the other experimental groups exhibited a significantly reduced spleen weight (Fig. 4E).

To elucidate the immune responses leading to the significant tumor suppression in mice prophylactically immunized with the nanovaccines, splenic lymphocytes were isolated and analyzed with flow cytometry. The frequency of CTLs (CD3^+^CD8^+^IFN-γ^+^) in splenocytes was significantly increased in the mice immunized with E7-NP compared to those immunized with the PBS control, MDP-NPs, β-glucan@ALG or MDP-NPs + β-glucan@ALG, although the latter three treatments produced significant elevations in the CD3^+^CD8^+^IFN-γ^+^ population in comparison with the PBS control. Notably, the nanovaccine E7- MDP-NPs + β-glucan@ALG more significantly increased the CD3^+^CD8^+^IFN-γ^+^ cell frequency than did the other treatments (Fig. 4F). To assess systemic immunosuppressive responses, the frequency of MDSCs (CD11b^+^Gr-1^+^) in splenocytes was further analyzed. Not surprisingly, MDSCs presented a contrary trend to CTLs. E7-MDP-NPs + β-glucan@ALG immunization produced the most significant decrease in the frequency of MDSCs (Fig. 4G). It is worth noting that the MDP-NP, β-glucan@ALG and MDP-NP + β-glucan@ALG groups also showed reduced CD11b ^+^ Gr-1^+^ cell responses in splenocytes compared to the PBS control group (Fig. 4G). The analysis of CD11b^+^Gr-1^+^ cells in local tumor tissues produced similar results (Fig. 4H).

### Therapeutic immunization with nanovaccines significantly promotes the antitumor immune response and suppresses or even abolishes established tumors

**Fig. 5:**
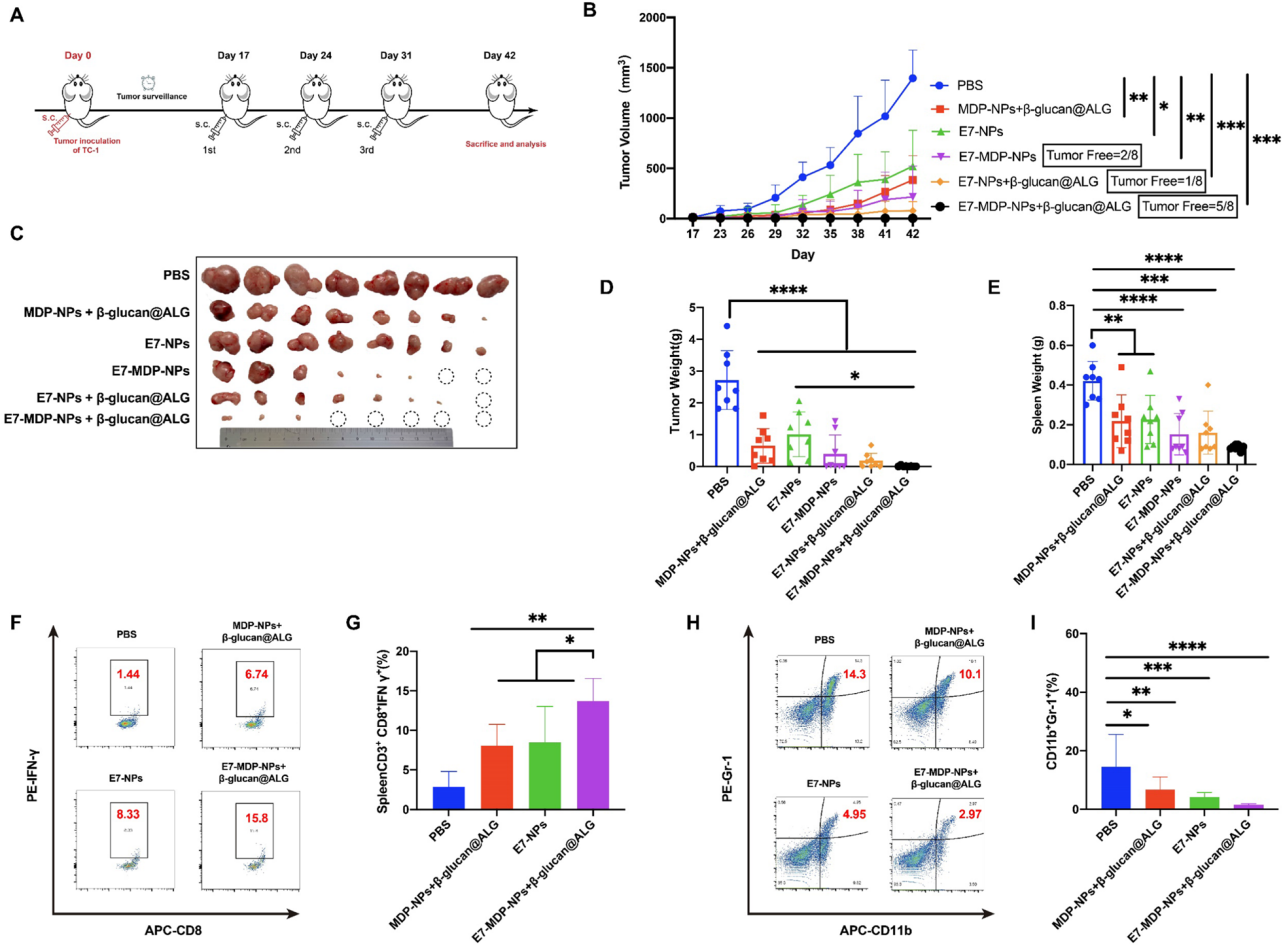
Therapeutic immunization with nanovaccines elicits significant antitumor effects and cellular immune responses. **A** Experimental protocol. The nanovaccines were administered when the size of grafted TC-1 tumors reached a diameter of 3 − 4 mm. n = 8 mice per group. **B** Dynamic monitoring of tumor growth. **C** Representative images of tumor masses at the experimental endpoint. **D** Tumor weight. **E** Isolated spleen weight. **F** and and **G** Statistical data for analyzing IFN-γ-expressing CD3^+^CD8^+^ splenocytes. The splenocytes were stimulated with the E7 peptide and analyzed by FACS analysis. **H** and **I** Statistical data for analyzing the frequencies of MDSCs (CD11b^+^ Gr-1^+^) in splenocytes. The data are shown as the mean ± standard error of the mean (SEM). The differences among groups were analyzed by one-way ANOVA with Tukey’s multiple comparisons test

To further evaluate the antitumor efficacy of immunization with the nanovaccines, a therapeutic experiment was performed, with immunizations conducted after grafted tumors were fully established (Fig. 5A). Dynamic monitoring of tumor growth, tumor size, and tumor weight showed that all the formulations significantly suppressed tumor growth compared to PBS. The nanovaccines consisting of E7-NPs and MDP, β-glucan and/or ALG hydrogel were able to completely abolish established tumors. In particular, the nanovaccine E7-MDP-NPs + β-glucan@ALG produced the most significant tumor inhibition compared with the other formulations (Fig. 5B–D). The spleen weight data were consistent with those for tumor size and weight (Fig. 5E).

The systemic response of key effector cells (CD3^+^CD8^+^IFN-γ^+^ cells) in E7-MDP-NP + β-glucan@ALG-immunized mice was more significantly increased than that in MDP-NP + β-glucan@ALG- or E7-NP-immunized mice when all groups were compared to the PBS control group (Fig. 5F, G). In addition, the frequency of immunosuppressive MDSCs (CD11b^+^ Gr-1^+^) in splenocytes was most significantly reduced in E7-MDP-NP + β-glucan@ALG-immunized mice (Fig. 5H, I).

It was indicated that the design of the two-phase delivery system with free β-glucan and encapsulated E7 and MDP in PLGA NPs further combined with the ALG hydrogel was extremely effective in eliciting antitumor cellular immunity and downregulating immunosuppressive cell responses, leading to significant suppression and even abolishment of established tumors.

### Nanovaccines stimulate trained innate immunity in vitro and in vivo

**Fig. 6:**
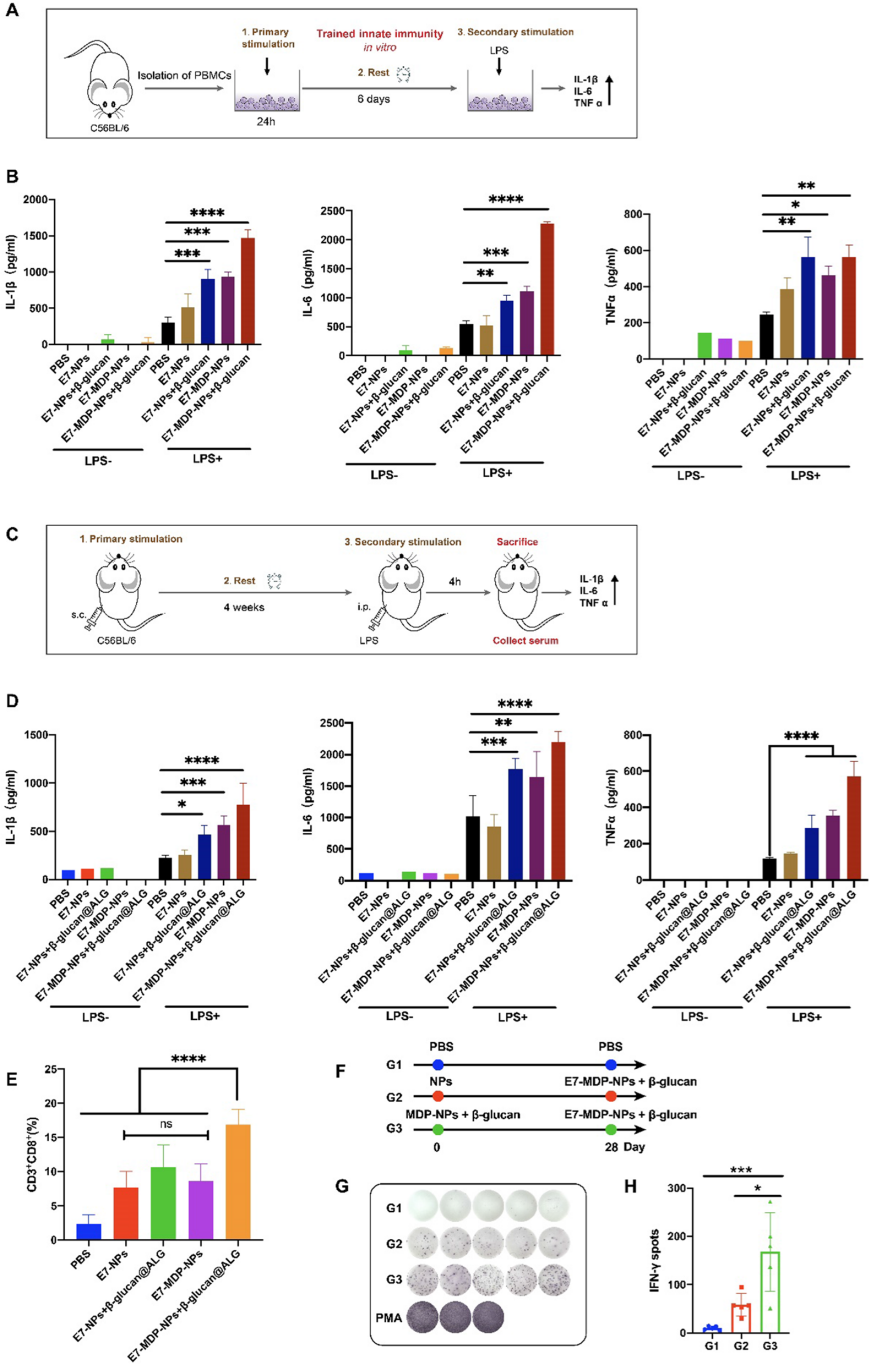
The nanovaccines induced trained innate immunity in vitro and in vivo. **A** Illustration of the in vitro training model. **B** Expression of the cytokines IL-1β, IL-6 and TNF-α after LPS restimulation. **C** Illustration of the in vivo training model. **D** Cytokine levels in serum after LPS restimulation. **E** FACS analysis of the frequencies of CD3^+^CD8^+^ cells in splenocytes. **F** Illustration of the experiment examining the effect of trained immunity on adaptive immune responses. **G** and **H** IFN-γ-expressing splenocytes determined by ELISpot. The data were analyzed by one-way ANOVA with Tukey’s multiple comparisons test

Both MDP and β-glucan are well-known “trained immunity" stimulators. To evaluate whether trained immunity can be induced by the prepared nanovaccines, in vitro (Fig. 6A) and in vivo (Fig. 6C) training experiments were performed. In the in vitro model, PBMCs were incubated with the different nanovaccine formulations, followed by heterologous stimulation with LPS and PBS, serving as a control, at a 6 day interval. The supernatants were analyzed for the levels of IL-1β, IL-6, and TNF-α produced by the PBMCs. The results showed that in comparison with the PBS control, which represented the response levels for these cytokines produced by PBMCs stimulated with LPS once, secondary stimulation with LPS significantly increased the cytokine levels in the groups receiving primary stimulation with E7-NPs + β-glucan, E7-MDP-NPs, or E7-MDP-NPs + β-glucan, with the largest increase in the E7-MDP-NPs + β-glucan group (Fig. 6B). The results clearly indicated that MDP and/or β-glucan in the nanovaccine formulations could train innate immunity, as evidenced by the boosted proinflammatory cytokine responses caused by the heterologous secondary stimulation, which indicated the “memory effects” of trained innate immunity.

Similar results were also shown in the in vivo training experiments (Fig. 6D). It is worth noting that in the in vivo experiment, the frequency of CD3^+^CD8^+^ immune cells in splenocytes in the E7-MDP-NPs + β-glucan group was significantly higher than that in the other groups (Fig. 6E), suggesting that the induction of trained innate immunity may promote antigen-specific antitumor adaptive cellular immune responses. An animal experiment was further employed to investigate whether the induction of trained immunity in advance is capable of affecting adaptive immunity responses elicited by a nanovaccine (Fig. 6F). The results showed that treatment with MDP-NPs + β-glucan, compared to treatment with NPs, significantly promoted the E7-MDP-NPs + β-glucan@ALG-stimulated response by E7-specific IFN-γ-expressing lymphocytes in the spleen (Fig. 6G, H), which was indicative of an antitumor cellular response.

## Discussion

The initiation and maintenance of adaptive T/B-cell responses and long-term protective memory cell responses are tightly associated with innate immunity. Trained innate immunity is characterized by “immune memory”, with a more robust innate immune response initiated upon recognition of secondary homologous or heterologous stimulators. It is quite attractive to introduce the concept of inducing “trained immunity” in vaccine design to help establish and maintain robust and long-lasting antigen-specific adaptive immunity.

Here, we developed a biphasic release system to facilitate the controlled delivery of a tumor antigen and the trained immunity agonists β-glucan and MDP. First, PLGA-based NPs were employed to encapsulate MDP and the specific tumor antigen E7 peptide to improve the stability and efficient intracellular delivery of these agents. Then, the PLGA NPs and β-glucan were further embedded into ALG to achieve sustained release of the NPs and β-glucan to provide efficient delivery to the lymph nodes and persistent stimulation of immune cells. For the induction of trained immunity, MDP was encapsulated in PLGA nanoparticles, aiming to deliver intracellularly to its intracellular receptor nucleotide-binding oligomerization domain 2 (NOD 2); and, β-glucan was just incorporated into hydrogel instead of encapsulated in PLGA nanoparticles to facilitate its diffusion and binding to the extracellular receptor dectin-1.

MDP is a common structural motif of peptidoglycan, with molecular weight: 492,48 g/mol, and derived from the wall of gram-positive and negative bacteria. In terms of structure, MDP consists of MurNAc and two amino acids, namely L-Ala and D-iGln. It is a glycopeptide that shows stimulatory effects on many functions of monocyte and macrophage, such as pinocytosis, phagocytosis, chemotaxis, and bactericidal and antitumor activity. We used a solvent evaporation two-stage emulsification method to prepare the W/O/W PLGA nanoparticles, which allows hydrophilic components such as MDP and E7_44-62_ peptides in this study to be encapsulated in the nanoparticles. Briefly, the components were dissolved in organic solvents, which were ultrasonicated with aqueous solution to form an emulsion. And then the organic solvents were removed through evaporation, which makes the dispersed phase solidified to form microspheres. PLGA nanoparticle is a classical large volume and erodible biopolymers, which are easily infiltrated into the matrix by water and form interstice, leading to the degradation of the particles. The encapsulated E7 or MDP can be released from the particles by this mechanism or by diffusion.

We first revealed that PLGA NPs containing β-glucan and MDP significantly improved the efficiency of antigenic peptide uptake by DCs (Fig. 2).It is generally accepted that NPs are more readily and efficiently recognized and taken up by antigen-presenting cells (APCs) due to their size, charge, and shape [48, 49] than the soluble antigen alone. The PLGA NPs in this study were between 100 and 300 nm in size, which is favorable for the migration and accumulation of antigens in the lymph nodes and uptake by APCs [50]. Our results suggested that β-glucan and MDP might directly affect APCs to improve their capacity to take up antigens encapsulated in NPs. Furthermore, we demonstrated that ALG was able to form a hydrogel in vitro upon interacting with calcium ions and in vivo in the injected site. Due to the multilayer porous structure and viscosity of the colloidal system of the formed hydrogel, β-glucan and PLGA NPs embedded in the ALG hydrogel achieved sustained release. Using an in vivo tracing experiment, we confirmed that ALG and PLGA NPs significantly increased the residence time of antigenic peptides at the injection site and promoted the accumulation of the antigen in the lymph nodes, which allowed the antigen to be taken up, processed, and cross-presented by more APCs and thus might lead to more efficient triggering of T- and B-cell responses. Our results also showed that β-glucan and MDP had the capability to promote DC maturation, which was evidenced by the increased expression of CD80, CD86, MHC II and MHC I. Our results are supported by previous studies [43] demonstrating that MDP derivatives act similarly to the BCG-PGN adjuvant in monocyte-derived DCs [51], showing agonistic activities on TLR2 and TLR4; in addition, β-glucan has been shown to promote DC maturation through the PI3K/Akt signaling pathway [52]. In our results, E7-MDP-NPs + β-glucan@ALG exhibited the strongest capability to promote DC maturation compared to the other formulations containing no agonist or only MDP or β-glucan.

MDP and β-glucan induce epigenetic changes on histones [53], leading to increased accessibility of the promoter regions of genes associated with inflammatory pathways. Thus, trained innate immune cells were endowed with a long-term capability to express higher levels of PRRs and proinflammatory cytokines and generate a faster and enhanced response to a secondary stimulus than untrained cells. To verify the training effects of the nanoformulations, in the current study, we adopted both an in vitro training model using isolated PBMCs and an in vivo model using mice based on previous reports [54, 55]. Compared to those for the corresponding controls that did not receive training, the results for both the PBMCs trained with the nanovaccines in vitro and serum isolated from mice receiving the training in vivo showed a stronger response with significantly higher levels of the proinflammatory cytokines IL-1β, IL-6, and TNF-α in response to LPS stimulation (Fig. 6), which indicated the successful induction of trained immunity [31, 56]. It is unclear whether different inducers train innate immune cells to produce the same functional behavior. However, different inducers may trigger different cell activation pathways and produce different modifications in cellular metabolism, affecting key cellular functions of trained innate immune cells [29]. It has been implied that different inducers or their combinations may induce different trained immunity outcomes, and antigens combined with different trained immunity inducers may produce varied characteristics in antigen-specific adaptive immune responses. So Our nanovaccine formulation E7-MDP-NPs + β-glucan@ALG produced the strongest training effects compared to the controls lacking the agonists or containing only MDP or β-glucan. Furthermore, we performed an experiment similar to the in vivo training model and directly unveiled that the induction of trained innate immunity significantly enhanced the responses of antigen-specific INF-γ-expressing immune cells elicited by subsequent stimulation with the nanovaccine, which implied the importance of inducing trained immunity through vaccine design to promote adaptive immune responses.

TC-1 tumor cells, which were obtained by cotransformation of HPV16 E6 and E7 and the activated ras oncogene into primary C57BL/6 mouse lung epithelial cells [42], which constitutively express HPV16 E6 and E7 proteins and present the MHC-peptide complex on the cell surface. Characterized by the HPV derived definite tumor antigens, TC-1 cells are frequently used to establish a tumor model in mice for assessing the potentials of a therapeutic vaccine for the treatment of HPV-related tumors. And, E7 peptide ^44^QAEPDRAHYNIVTFCCKCD^62^ was identified as an important T cell epitope in C57BL/6 mice, and thus is widely used as a representative antigen for vaccine construct and evaluation in TC-1 tumor model. In a preventive immunization strategy, nanovaccine administration significantly inhibited the growth of subsequently grafted TC-1 tumors. All mice receiving E7-MDP-NPs + β-glucan@ALG failed to develop tumors (Fig. 4). Mechanistically, E7-MDP-NPs + β-glucan@ALG significantly promoted tumor-specific cell-mediated immunity, with a significantly increased frequency of E7 peptide-specific CD3^+^CD8^+^IFN-γ^+^ cells and a reduced frequency of immunosuppressive MDSCs in the spleen and tumor. Interestingly, mice receiving MDP, β-glucan or both agonists demonstrated significantly suppressed tumor growth in the absence of additional specific tumor antigen, although the inclusion of the specific antigen in the formulation further significantly enhanced the antitumor effects.

An immunosuppressive tumor microenvironment (TME) is established through the recruitment of immunosuppressive cells, including tumor-associated macrophages, tolerant or immature DCs, regulatory T cells (Tregs), and MDSCs, which render the tumor resistant to immunotherapeutics [57]. Therefore, combining the modulation of the TME toward an immunoresponsive state with immunotherapy has the potential to enhance the antitumor effects of the immunotherapeutic strategy. β-glucan and MDP have been shown to be effective adjuvants [23, 58] and have the capacity to modulate the TME [59, 60]. β-glucan is well tolerated even at a high dose (up to 10 mg/kg), contains many side groups that allow further functional modification, and has been used as an immunoadjuvant for cancer treatment as a single agent or in combination with other agents [59]. MDP, as a NOD agonist, also acts as an immunotherapeutic agent. While stimulation of NK cells [61] and DCs [62] has been reported, the predominant activation of monocytes and macrophages by MDP has also been demonstrated [23]. Pretreatment of mice with β-glucan results in reduced tumor growth due to the antitumor effects of β-glucan-induced trained immunity on granulocytes and neutrophils producing TME reprogramming to an antitumor phenotype through transcriptomic and epigenetic remodeling. This process requires type I interferon signaling, which is independent of host adaptive immunity [23]. In summary, MDP and β-glucan can play immunomodulatory roles in antitumor responses in the TME or immune organs by inducing trained immunity, which may address the tumor suppression found in mice receiving MDP and β-glucan without the E7 antigen in this study.

In a treatment experiment, nanovaccine immunization was performed after grafted tumors were fully established (diameter of 2–4 mm). Encouragingly, the E7-MDP-NPs + β-glucan @ALG vaccine significantly inhibited the growth of established tumors, with 5 of 8 mice becoming completely tumor free, demonstrating the notable capacity of immunization with this nanovaccine to reverse tumor growth kinetics and even regress established tumors. Similar to the findings in the preventive study, MDP-NPs + β-glucan@ALG or E7-NPs alone also significantly suppressed tumor growth, while the combined use of MDP or β-glucan with E7-NPs produced more robust antitumor effects.

We indicated that the enhanced responses of trained innate immune cells may promote tumor antigen-specific adaptive cellular immunity in this study, providing a promising strategy of developing an effective vaccine. The safety profile of employing trained immunity inducers to serve as vaccine adjuvants is supported by the facts that the attenuated vaccines such as BCG, OPV, and measles vaccines, which are successfully applied in human clinic with a long history, are capable of inducing trained immunity; recently, trained immunity were induced with BCG in clinical trials for assessing its potentials of providing a broad protection against SARS-COV-2; in addition, it has been found that some successful clinical adjuvants cause trained immunity. However, trained immunity can also be detrimental in some disease settings. In principle, excessive or chronic activation of innate immune responses by inducing trained immunity could lead to excessive inflammation or immunosuppression during chronic disease [63]. A growing body of research provides evidence that the reprogramming of trained immunity may play a role in the maintenance of a variety of diseases. It should be noted that too strong or continuous trained immunity may not be a good thing, for example, atherosclerosis and some autoimmune diseases may be related to continuous trained immunity [64–66]. And thus, how to grasp the strength and durability of inducing trained immunity may be a primary issue to be addressed by the future deeper researches before the clinical application of “trained immunity”-based vaccines comes true.

## Conclusions

This study developed a biphasic release system-based antitumor nanovaccine, which featured controlled release and targeted cellular delivery of trained immunity agonists and a specific tumor antigen. The results indicate that the nanovaccine formulation has the abilities to induce trained innate immunity and elicit powerful antitumor adaptive cellular immunity, significantly reversing tumor growth and even completely eliminating established tumors. The study proposes that the induction of trained immunity may provide a new idea for developing a promising tumor vaccine.

## Data Availability

The data used and/or analyzed to support the current study are available from the corresponding author on reasonable request.
